# Commentary on: physical activity and exercise for mild cognitive impairment and dementia: a collaborative international guideline

**DOI:** 10.1007/s40520-024-02747-1

**Published:** 2024-04-23

**Authors:** Nicola Veronese, Pinar Soysal, Mario Barbagallo, Susan D. Shenkin, Terence J Quinn

**Affiliations:** 1 Geriatric Unit, Department of Internal Medicine and Geriatrics, University of Palermo, Via del Vespro, 141, Palermo, 90127 Italy; 2 Division of Geriatric Medicine, Faculty of Medicine, Bezmialem Vakif University, Istanbul, Turkey; 3 Ageing and Health, Usher Institute, Edinburgh University, Edinburgh, UK; 4 NIHR Evidence Synthesis Group @Complex Review Support Unit, School of Cardiovascular and Medical Sciences, College of Medical, Veterinary and Life Sciences, University of Glasgow, Glasgow, UK

**Keywords:** Dementia, Mild cognitive impairment, Guidelines, Physical activity

## Abstract

Physical inactivity is an important, but potentially reversible risk factor for dementia and mild cognitive impairment (MCI). There is literature about physical activity and exercise for the prevention and management of dementia and MCI, but this had not been previously synthesized into specific guidelines about this topic. A recent guideline on physical activity and exercise in MCI and dementia was published, authored by several international societies, including lay representatives. In this commentary, we discuss the implications of this guidance for healthcare professionals, caregivers, and lay representatives involved in the care of people with MCI and dementia.The guidelines highlight the importance of physical activity and exercise in cognitively healthy persons and for dementia and MCI, at different stages of these conditions. For primary prevention of dementia, physical activity may be suggested in cognitively healthy persons. In people with MCI, mind-body interventions, such as yoga, have the greatest evidence, whilst the role of physical activity and exercise requires more evidence from high-quality randomized controlled trials. In people living with moderately severe dementia, exercise may be useful for maintaining physical and cognitive function. There are benefits of physical activity and exercise separate from their impact on cognitive outcomes. The guidelines also proposed some questions for future research. In conclusion, there is limited evidence on the beneficial role of physical activity and exercise in preserving cognitive functions in subjects with normal cognition, MCI or dementia. The guidelines support the promotion of physical activity based on the beneficial effects on almost all facets of health.

## Introduction

Dementia is one of the most pressing global public health priorities. In the absence of proven disease modifying or reversing interventions, attention has turned to dementia prevention. The 2020 Lancet commission proposed twelve modifiable risk factors that may account for 40% of global dementia burden [1]. The Lancet Commission has influenced research and policy, but arguably direct, person level interventions are still lacking.

A potentially important modifiable risk factor identified by the Commission was levels of physical activity. It is estimated that increasing physical activity in middle-aged and older people could prevent between 3% and 18% of all cases of dementia observed [2–5]. Physical activity in this context could include both physical activity (i.e., any bodily movement produced by skeletal muscles that results in energy expenditure) and exercise (i.e., a subset of physical activity that is planned, structured, and repetitive), as both have plausible data to suggest a role in preventing or slowing the pathological processes that underly dementia syndromes [6] as well as having widespread beneficial effects on other conditions (e.g., cardiovascular health, diabetes, cancer) [7]. 

In spite of a large body of published science regarding physical activity and cognition, interpretations have been conflicting and there has been debate around applying the evidence to specific populations. In this situation, evidence based international guidelines, offering practical advice to busy health and social care professionals and others affected by dementia is especially welcome. In this commentary, we discuss the evidence on how physical activity and exercise affects cognition – specifically differentiating the prevention and management of both Mild Cognitive Impairment (MCI) and dementia - in the context of a recently published guideline derived from the work of several international scientific societies and lay representatives [8]. 

### Benefits of physical activity and exercise in older people

A case for promoting exercise in older adults seems plausible for a wide range of reasons. Observational data suggest that increasing physical activity can benefit chronic medical conditions [9], while insufficient physical activity level is associated with important geriatric syndromes, such as sarcopenia, frailty, and disability [9]. Similarly, a recent meta-analysis showed that every step counts: in 17 cohort studies including almost 250,000 participants, an increase in 1000 steps/daily was associated with a decrease in mortality risk of 15%, mainly driven by a decrease in cardiovascular events [10]. Beyond cardiovascular disease, where increasing activity should bring benefits mediated by improved fitness and effects on blood pressure and cardiac output [11], exercise also seems to benefits people living with psychiatric disorders, such as depression. A recent network meta-analysis including 21 randomized controlled trials (RCTs) and 2551 participants, the found that exercise was as effective as pharmacological interventions in reducing depressive symptoms [12]. 

Finally, regular physical activity and exercise play a crucial role in maintaining and improving functional capacity and independence in older people. Engaging in physical activity, such as aerobic exercise, strength training, and balance exercises, has been shown to enhance muscle strength, flexibility, cardiovascular health, and overall mobility [13]. These benefits translate into improvements in activities of daily living (ADLs), such as walking, climbing stairs, and performing household tasks, ultimately preserving functional independence and quality of life in older adults [14]. 

### Physical activity and exercise for cognition: the need for a new guideline

With so much data to support the benefits of exercise, one could ask why a specific guideline with a cognition focus was required. Firstly, the bulk of the data available and that inform other guidelines are from observational studies that sometimes did not include people with cognitive disorders [15, 16]. While useful, such data are prone to numerous biases and creating recommendations based on these data comes with several caveats. Secondly, we should not assume that generic guidelines, tailored for people without cognitive issues, will be appropriate to people at risk of, or living with, cognitive problems, since the translation of the indications proposed for generally healthy people could be hard to understand for people with dementia or MCI. Thirdly, we recognize a potential evidence-practice disconnect. Several guidelines regarding the importance of physical activity/exercise are available [15, 16]. The World Health Organization (WHO), for example, recommend targets for older people of 150 min of moderate- or 75 min of vigorous-intensity aerobic activity, along with two or more days of muscle-strengthening activity (i.e., strength/resistance training) per week [15]. However, these suggestions are not linked to cognitive aspects [15]. Fourthly, although data specific to cognition are available, for example as reported in the comprehensive umbrella review regarding the topic [17], guidelines, based on a systematic approach to the literature and specific for people living with dementia, mild cognitive impairment and other cognitive syndromes are still not available.

### Development and content of the joint guideline on physical activity and cognition

These collaborative guidelines, involving nine different societies, were published in the middle of August 2023 [8]. 

The literature searching was carried out by two expert librarians using several databases, from databases’ inception to 09th October 2021 for systematic reviews with or without meta-analysis about the role of physical activity/exercise for the prevention and management of MCI and dementia.

The writing group was formed by the Presidents of each society (or a representative), the Chairperson for the Guidelines (appointed by the European Geriatric Medicine Society, EuGMS), five experts who were involved as chairs of the topics of the guidelines, i.e., the role of physical activity/exercise in primary prevention, MCI, and dementia, respectively. The literature was reviewed and graded using the GRADE (Grading of Recommendations Assessment, Development and Evaluation). The experts, the chairperson of the guidelines, and one expert of each society not previously involved in the manuscript drafting discussed the recommendations during an online meeting (01st April 2023) and agreement was reached through discussion. Consensus on each question/intervention was defined if at least 80% of the members of the working group were either “strongly” or “weakly” in favor or against a recommendation [14]. 

**Fig. 1:**
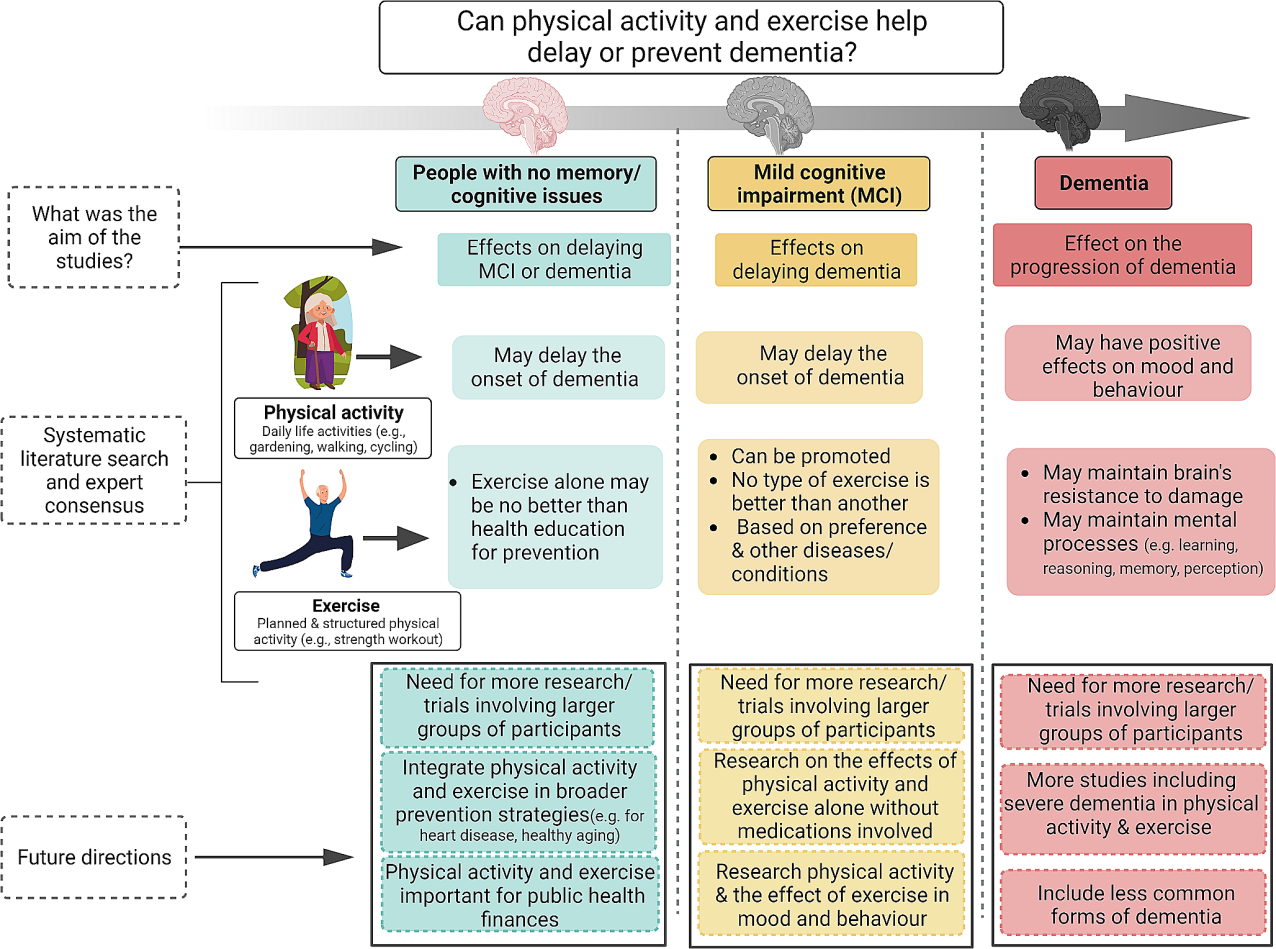
Infographic about the role of physical activity and exercise in the prevention and management of mild cognitive impairment and dementia

The main recommendations in the guidance, as summarized in the infographic (Fig. 1) are:

1. In people without any evidence of dementia or MCI, physical activity may be considered for the primary prevention of dementia, including both AD, or vascular dementia subtypes, albeit the certainty of the evidence was very low;
2. In people living with MCI, there is continued uncertainty about the role of physical activity and exercise in slowing the conversion to dementia. Based on low quality evidence, mind-body interventions seemed most effective in improving cognitive outcomes, whilst the role of aerobic and anaerobic exercise should be explored by future trials.The cautious recommendations around MCI are based on the studies considered with highly heterogeneous interventions, small sample size, and surrogate outcomes rather than considering conversion to dementia.
3. In people living with moderate dementia, physical activity/exercise could be considered for maintaining cognition and stabilizing disability and improving neuropsychiatric symptoms (in particular depression) and the risk/number of falls.In planning the guideline, there were topics we wished to cover but were ultimately unable to offer guidance as even with comprehensive, contemporary systematic reviews and meta-analyses, the questions remain unanswered. For example, we were not able to give recommendations regarding the optimal “dose” of physical activity/exercise, as in the included studies, specific details on the physical activity/exercise intervention (type, frequency or intensity) were poorly reported. While our intention was to offer tailored recommendations for dementia subtypes and stages, available trial data were mostly limited to moderate severity Alzheimer’s disease. Finally, we were keen to look at person-centered outcomes such as quality of life or caregiver stress, but these data were not available [8].

In our guidelines, among the recommendations of research, we underline that (a) future RCTs should investigate the role of physical activity and exercise in the conversion of MCI to dementia; (b) the inclusion of a range of doses/types of physical activity; (c) the inclusion of people living with a range of dementia subtypes and severity; (d) the inclusion of person-centred outcomes; (e) consideration of role of technology, and (f) strategies to increase physical activity with a person-centred approach.

### Strategies for increasing physical activity levels in older people in mild cognitive impairment and dementia

In these guidelines, we considered issues around implementing our physical activity recommendations. Factors that seem to influence physical activity among older people with MCI or dementia, include resources available, social support, and perceived competence [18]. Using behavior change techniques, we propose six implementation strategies: providing supervision, developing tailored interventions, providing safe and promoting environment, helping to increase participants’ motivation and adherence, integrating all kinds of social support, and providing suitable staffing [18]. We believe also that technologies could have potential to increase physical activity level in older people, even if, at present, there is low to moderate evidence that interventions delivered via technological approaches are effective in increasing physical activity in older adults [19]. 

## Conclusions

We hope that these guidelines will increase the awareness of health and social care professionals, as well as people living with dementia and their families, of the importance of physical activity and exercise for the prevention and management of cognitive disorders. While the supporting evidence was imperfect, we believed it was sufficient to make a strong recommendation around the use of physical activity and exercise for the prevention and management of MCI and dementia. Finally, while it is likely that exercise sustained over decades may prevent the onset of dementia and MCI, research is ongoing to explore whether exercise significantly changes the course of dementia once it is established. In this direction, some recent RCTs have reported that in early dementia/MCI stage exercise was not better than usual care to improve cognitive outcomes [20, 21]. Therefore, today, the main goal of literature about exercise in cognition is to maximize other benefits, such as cardiovascular ones, that are supported by a more solid literature. We hope that this unambiguous recommendation will influence research, practice and policy.

## Data Availability

No datasets were generated or analysed during the current study.
